# Multiple genome sequences of lactic acid bacteria, *Carnobacterium divergens* and *Carnobacterium maltaromaticum* strains, isolated from vacuum-packaged lamb meat

**DOI:** 10.1128/mra.01058-23

**Published:** 2024-01-10

**Authors:** Faith Palevich, Amanda Gardner, Colleen Ross, John Mills, Gale Brightwell, Nikola Palevich

**Affiliations:** 1 Food System Integrity, Hopkirk Research Institute, AgResearch Ltd, Palmerston North, New Zealand; 2 Rumen Microbiology, Grasslands Research Centre, AgResearch Ltd, Palmerston North, New Zealand; Portland State University, Portland, Oregon, USA

**Keywords:** *Carnobacterium*, lactic acid bacteria, genomes

## Abstract

Here, we report the whole-genome sequences of 11 *Carnobacterium divergens* and 2 *Carnobacterium maltaromaticum* bacteria isolated from vacuum-packed chill-stored lamb meat in New Zealand. Examination of these lactic acid bacteria (LAB) genomes will improve our knowledge of their potential antimicrobial activities and spoilage mechanisms of importance to the meat industry.

## ANNOUNCEMENT

*Carnobacterium divergens* and *Carnobacterium maltaromaticum* are Gram-positive, psychrotrophic lactic acid bacteria (LAB), associated with spoilage of long shelf-life vacuum-packed (VP) meat and seafood after prolonged storage at chilled temperatures (1, 2). *Carnobacterium* species also display antimicrobial properties through the production of bacteriocins to extend the shelf life of meat products (3, 4).

Here, we report the whole-genome sequences of 11 *C. divergens* and 2 *C. maltaromaticum* strains isolated from VP chilled lamb samples obtained from a commercial abattoir in New Zealand (-40° 21′ 22.90″S, 175° 36′ 40.07″E). Briefly, meat samples were homogenized with maximum recovery diluent broth (BD Difco) using a stomacher (Seward), plated onto M5 agar (Fort Richard Laboratories), and incubated at 25°C for 3 days. Pure isolates were subcultured onto APT agar (Fort Richard Laboratories) and colonies were transferred into brain heart infusion broth (Bacto) with 15% glycerol.

Genomic DNA was extracted using a modified phenol-chloroform procedure (5) from cultures grown on 10 mL LB medium (Fort Richard Laboratories) for 5 days at 25°C anaerobically. DNA concentration was determined using NanoDrop ND-1000 (ThermoFisher Scientific) and Qubit Fluorometer dsDNA BR Kit (Invitrogen), while DNA integrity was evaluated using gel electrophoresis. The 16S rRNA gene was amplified using AmpliTaq Gold 360 master mix (Applied Biosystems) with pA and pH primers (6), Sanger sequenced on an ABI 3730 DNA sequencing system (Applied Biosystems), and alignment to the NCBI 16S rRNA database using BLAST v2.9.0 (7).

Multiple DNA libraries were prepared using the NuGEN Celero PCR workflow with enzymatic fragmentation DNA library preparation kit according to the manufacturer’s protocol and sequenced using the Illumina MiSeq (2 × 250 bp) v2 platform to generate 12 to 15 million paired-end reads. Read quality was evaluated using FastQC v0.12.0 (8) and trimming of raw sequences was performed using Trimmomatic v0.39 (9). The A5-miseq pipeline v20169825 (10) was used for the *de novo* assembly of the genomes. The contigs for each strain were annotated using NCBI’s Prokaryotic Genome Annotation Pipeline (PGAP) v6.6 (11). CheckM v1.2.2 (12) was used to estimate genome completeness and contamination using the *Carnobacteriaceae* marker set. Default parameters were used for all software unless otherwise specified. Features of the 13 *Carnobacterium* strains are summarized in Table 1.

**TABLE 1:** Genomic features and accession numbers for the 13 *Carnobacterium* strains^*d*^

Metadata	*C. divergens*											*C. maltaromaticum*	
Strain identifier^a^	9-8	9-12	9-13	9-14	9-47	9-48	9-49	9-68	9-69	9-70	12-22	12-15	12-25
Isolation source (cut of lamb)^b^	Shank	Loin	Loin	Shank	Loin	Loin	Shank	Loin	Loin	Loin	Shank	Loin	Shank
Lamb storage temperature (°C)	−1.5	−1.5	−1.5	−1.5	0	0	2	2	2	2	−1.5	−1.5	0
**Genome statistics**													
Assembly size (bp)	2,816,712	2,718,778	2,719,987	2,896,130	2,632,303	2,631,423	2,630,737	2,758,961	2,672,549	2,718,926	2,895,279	3,643,485	3,646,517
Genome coverage (×)	124	90	141	112	141	92	136	78	170	134	102	74	79
G + C content (%)	35	35.5	35.5	35	35.5	35.4	35.5	35	35.5	35.5	35	34.5	34.5
No. of paired reads (2 × 250 bp)	1,001,970	739,631	1,174,388	928,511	1,081,249	745,641	1,043,480	809,873	1,230,878	1,164,847	839,921	842,582	885,546
No. of contigs	20	28	33	53	24	23	21	52	29	28	52	33	39
Contig N50 (bp)	400,178	387,520	379,750	170,520	387,520	387,603	394,883	214,322	387,420	496,650	165,419	786,356	525,136
Contig L50	2	2	2	6	2	2	2	6	2	2	7	2	3
Completeness (%)	98.22	98.22	98.22	98.22	98.22	98.22	98.22	98.22	98.22	98.22	98.22	98.22	98.22
Contamination (%)	5.45	4.79	4.79	4.7	4.52	4.52	4.52	5.28	4.69	4.79	4.7	11	11
**Genome annotations (RefSeq)**													
No. of genes	2,730	2,660	2,656	2,835	2,549	2,545	2,546	2,767	2,606	2,666	2,836	3,473	3,484
No. of CDS	2,651	2,548	2,546	2,744	2,455	2,452	2,451	2,667	2,506	2,550	2,754	3,352	3,356
No. of rRNAs	15	19	21	13	19	18	19	17	21	17	15	19	23
No. of tRNAs	52	60	56	56	56	56	57	56	60	66	47	66	68
**Genome project information**													
GenBank ID	JALRMQ01	JALRNA01	JALRMP01	JALRMR01	JALRMS01	JALRMT01	JALRMU01	JALRNB01	JALRMV01	JALRMW01	JALRMY01	JALRMX01	JALRMZ01
BioSample ID (SAMN)	27571581	27571591	27571580	27571582	27571583	27571584	27571585	27571592	27571586	27571587	27571589	27571588	27571590
Assembly ID (ASM)	31921525	31921365	31921565	31921485	31921545	31921505	31921465	31921285	31921405	31921445	31921425	31921385	31921325
SRA ID (SRR)	19151059	19151057	19151060	19151055	19151054	19151053	19151052	19151056	19151051	19151050	19151048	19151049	19151058
**Taxonomy (best match genome)** ^**c**^													
*C. divergens* DSM 20623^T^	99.02|91.2	93.7|52.7	93.71|52.7	99.03|91.2	93.7|52.7	93.7|52.7	93.7|52.7	99.06|91.8	93.7|52.7	93.7|52.7	99.03|91.1	-	-
*C. maltaromaticum* DSM 20342^T^	-	-	-	-	-	-	-	-	-	-	-	99.16|91.8	99.15|91.9

^*a*^ Strains starting with 9- and 12- indicate nine- and twelve-weeks storage, respectively.

^*b*^ pH of lamb cuts was different for loin (pH 5.6-5.8) and shank (pH 6.2-6.4).

^*c*^ % identity between the query genome and best match type strain from the NCBI Taxonomy Database and DSMZ collection (ANI | dDDH d_4_). GenBank accession numbers for *C. divergens* DSM 20623^T^ 16S rRNA gene (NR_044706) and genome assembly (GCA_001437085); *C. maltaromaticum* DSM 20342^T^ 16S rRNA gene (NR_044710) and genome assembly (GCA_000744945). -, indicates dDDH *d_4_* values below <25%.

^*d*^ Abbreviations: ANI, average nucleotide identity; CDS, protein coding sequences; dDDH *d_4_*, proportion of sequence identity matched between genome and the most similar reference genome.

Taxonomic assignments of our strains were explored using NCBI’s average nucleotide identity (ANI) workflow (13) at genome submission and *in silico* digital DNA-DNA hybridization (dDDH) analysis *via* the Type (Strain) Genome Server (TYGS) v387 (14). The combined phylogenetic analyses of the 16S rRNA gene sequences, ANI and dDDH (*d*_*4*_) value analyses revealed the best-match and closest relatives were *C. maltaromaticum* DSM 20342^T^ (15) and *C. divergens* DSM 20623^T^ (16) type strains, with 7 *C. divergens* strains constituting putatively novel species (17).

Genome-wide identification of secondary metabolite biosynthesis gene clusters (BGCs) was predicted using antiSMASH v7.0 in --strict mode option (18) and BAGEL v.1.2 webserver (19). Strong hits for two-peptide lantibiotic (carnolysins A1 and A2) were revealed for *C. maltaromaticum* strains; however, their presence warrants further detailed investigation. The reported *Carnobacterium* genomes serve as a valuable resource to explore their phylogenetic positioning, spoilage mechanism, and strain-level biopreservative potential.

## Data Availability

The genome sequences and associated data for the *Carnobacterium* strains have been deposited under the GenBank BioProject accession number PRJNA574489. Detailed information regarding GenBank, BioSample, Assembly, and Sequence Read Archive (SRA) accession numbers can be found in Table 1.
